# Clinical and Technical Characteristics of Coronary Angiography and Percutaneous Coronary Interventions Performed before and after Transcatheter Aortic Valve Replacement with a Balloon-Expandable Valve

**DOI:** 10.1155/2019/3579671

**Published:** 2019-08-08

**Authors:** Alfredo Nunes Ferreira-Neto, Rishi Puri, Lluis Asmarats, Victoria Vilalta, Leonardo Guimaraes, Robert Delarochellière, Jean-Michel Paradis, Siamak Mohammadi, Eric Dumont, Josep Rodés-Cabau

**Affiliations:** Quebec Heart & Lung Institute, Laval University, Quebec City, Quebec, Canada

## Abstract

**Objectives:**

To report on the feasibility and technical differences between coronary procedures performed before and after TAVR with the balloon-expandable Edwards-SAPIEN or the SAPIEN XT valves.

**Background:**

Coronary artery disease (CAD) and aortic stenosis often coexist. Transcatheter aortic valve replacement (TAVR) is emerging as a treatment for younger and lower surgical risk patients who might not present with clinically evident CAD before TAVR. The demand for performing post-TAVR coronary angiograms (CAs) and percutaneous coronary interventions (PCIs) will thus increase, posing new technical challenges.

**Methods:**

Over 1000 TAVRs were performed at the Quebec Heart and Lung Institute, of which 616 with the abovementioned valves. Of these, 28 patients had an analyzable pre- and post-TAVR CAs and 13 patients had pre- and post-TAVR PCIs performed. Procedural characteristics were gathered from all coronary procedures and subsequently compared amongst the same type of procedure performed at these two distinct time periods.

**Results:**

Neither CAs‐nor PCIs‐performed after valve implantation revealed significant differences regarding arterial access site, catheter diameter, number of diagnostic or guiding catheters used, procedural duration, fluoroscopy time, or achievement of selective coronary injection. Lesion location and classification, as well as the preference of using a drug-eluting stent, remained unchanged. During post-TAVR CA, the amount of contrast delivered and the radiation dose area product were significantly lower compared with pre-TAVR CA values.

**Conclusions:**

Performance of CA and PCI after TAVR with a balloon-expandable valve appears unaffected by its presence.

## 1. Introduction

There is considerable overlap in both the pathophysiology and presence of atherosclerotic coronary artery disease (CAD) and calcific aortic stenosis (AS), with concomitant significant CAD observed in up to 50% of individuals presenting with severe AS [1]. Given the establishment of transcatheter aortic valve replacement (TAVR) and its inevitable expansion towards the treatment of younger and lower surgical risk AS patients [2], the likelihood of encountering CAD remotely after TAVR will increase. The need for post-TAVR coronary angiography (CA) or percutaneous coronary intervention (PCI) was thus far relatively rare amongst initial TAVR cohorts who were deemed either inoperable or at high-to-extreme surgical risk. These individuals often succumbed in the post-TAVR period to their burdensome comorbidities [3]. The present era, however, represents a period in TAVRs' evolution whereby the incidence for the need to perform CA and possible PCI after TAVR has considerably increased, posing new technical challenges for the coronary interventionalist.

Transcatheter heart valves (THV) can be broadly divided into balloon-, self- or mechanically expanding in nature [4], with each prosthetic class possessing unique characteristics pertaining to their design, material, annuloaortic orientation, and anatomic relation to the native coronary tree. These THV design differences may thus impact on how coronary interventionalists access the native coronary vasculature. Despite various reports describing technical differences and potential challenges of CA and PCI in TAVR recipients [5], the nuances and technicalities for catheterizing and effectively accessing the native epicardial coronary vasculature in the presence of *in situ* THV are yet to be systematically reported in the presence of a balloon-expandable valve system. The present analysis was thus aimed at reporting the feasibility and potential challenges faced when performing coronary procedures after TAVR with either the balloon-expandable Edwards-SAPIEN or the SAPIEN XT THVs.

## 2. Materials and Methods

### 2.1. Study Population

**Figure 1:**
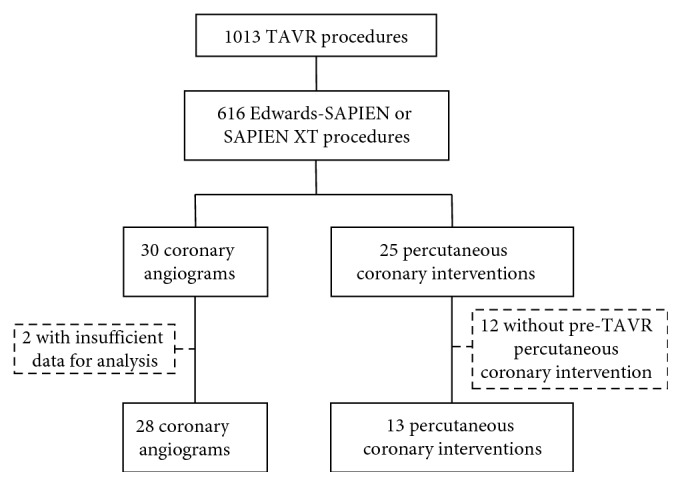
Flowchart of the study population.

Figure 1 illustrates a detailed flowchart describing the selection process implemented to obtain the intended cohort of patients for this study. Over 1000 TAVRs were performed in the last decade at the Quebec Heart and Lung Institute, of which 616 patients had either an Edwards-SAPIEN or a SAPIEN XT THV implanted. From these patients, a total of 68 coronary procedures, either a CA or PCI, were achieved after TAVR. From a total of 30 post-TAVR CAs performed, 2 CAs had insufficient data for complete analysis and were thus excluded, leaving a total of 28 patients available for analysis. Twenty-five patients underwent a PCI post-TAVR, but only 13 of these also had a pre-TAVR PCI with sufficient data to permitting complete analysis. All patients who presented with a combination of either pre- and post-TAVR CA (*n* = 28) or pre- and post-TAVR PCI (*n* = 13) ultimately comprised the present study's population.

### 2.2. Coronary Angiograms and Percutaneous Coronary Interventions

Coronary angiograms were mandatory for all patients before TAVR. However, post-TAVR angiograms were requested by either the attending cardiologist responsible for each patient's clinical follow-up or by an emergency department cardiologist where patients were admitted. Percutaneous coronary interventions were executed or deferred prior to TAVR as decided by either the patients' primary cardiologist, the Heart Team (comprising of at least 1 interventional cardiologist and acardiac surgeon), or by an interventional cardiologist performing the CA in the setting of an acute coronary syndrome (ACS) before TAVR. The need for post-TAVR PCI was evaluated in a similar fashion to post-TAVR CA. Neither the CA nor the PCI performed before or after TAVR had to be necessarily performed by the same interventional cardiologist; however, all analyzed procedures were performed by an experienced interventional cardiologist from the same tertiary high-volume institution in which the TAVR procedures occurred. In fact, the interventional cardiologists responsible for the procedures assessed in the present study have had several years of experience as interventionalists, performing about 400 coronary angiograms and 250 percutaneous coronary interventions per interventionalist/year. All pre- and post-TAVR procedures were reviewed by two experienced interventional cardiologists, and all relevant procedural and clinical characteristics were retrospectively gathered in a designated database for detailed analyses. The present study had no intention of comparing angiography to PCI, but rather to compare the same class of procedure performed in each patient before and after TAVR, since the comparison of technical aspects from two differing procedures might result in misleading findings.

### 2.3. Statistical Analysis

Two different sets of analyses were implemented in the present study, since 2 distinct groups were designed, one being the angiography group, in which only patients who had isolated CAs before and after TAVR were included, and the other being the PCI group, comprising patients who had PCI before and after TAVR. Categorical variables were assessed through the McNemar test and reported as absolute frequencies and percentages while continuous variables were analyzed using the Wilcoxon signed-rank sum test and are presented as mean ± SD. Results were deemed significant when a 2-sided *p*-value below 0.05 was achieved. The Statistical Package for Social Sciences version 24 (SPSS Inc., IBM, Armonk, New York) was used to conduct all the statistical analysis.

## 3. Results

**Figure 2:**
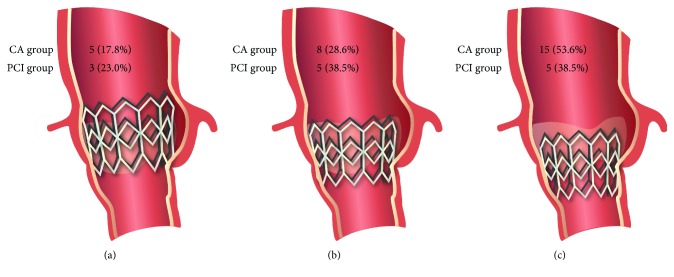
Diagram of the balloon-expandable transcatheter heart valve (THV) in regard to the coronary ostia. (a) Supra-ostial position of the THV (superior border of the stent frame above the lowest coronary artery ostia). (b) THV at the ostial level (superior border of the stent frame at the same level as the lowest coronary artery ostia). (c) Infra-ostial position of the THV (superior border of the stent frame below the lowest coronary artery ostia).

Table 1 describes baseline clinical characteristics and the TAVR procedural data for both the angiography and the PCI group. The presence of CAD was defined as prior coronary revascularization (percutaneous or surgical), prior myocardial infarction, or known coronary stenosis of at least 50% in diameter by visual evaluation. The THV position with respect to the coronary ostia is shown in Figure 2.

### 3.1. Coronary Angiograms

Post-TAVR CAs were performed at a mean time of 748 ± 686 days. The analyses of all aspects from the performed coronary angiograms, as seen in Table 2, showed that post-TAVR CA was more frequently performed in ACS scenarios, mainly due to unstable angina. The vast majority of procedures were performed through a radial approach (67.9% pre-TAVR, 64.3% post-TAVR, *p*=0.602).

Prior to TAVR, a mean of 1.21 ± 0.69 catheters/per patient was needed to cannulate the left coronary artery (LCA) which was most commonly (89% of cases) achieved with a Judkins left (JL) catheter (Cordis Corporation, Freemont, CA, USA). In cases where cannulation was unachievable or deemed inappropriate, other catheters such as the Multipurpose (MP) or the Amplatz Left (AL) diagnostic catheters (Cordis Corporation, Freemont, CA, USA) were used as substitutes to the JL. A mean of 1.21 ± 0.79 catheters/per patient was used to perform the right coronary artery (RCA) angiogram before TAVR, and in 82% of cases, the Judkins Right (JR) catheter was used, while the Amplatz Right (AR) or the Barbeau diagnostic catheter were the most commonly used surrogates in case of failure with the first catheter. Noticeably, the implanted THV did not result in any increase in the number of catheters needed to perform CA after TAVR (1.04 ± 0.33 catheters for the LCA and 0.96 ± 0.58 catheters for the RCA). Furthermore, the preferred choices of diagnostic catheters did not change significantly post-TAVR, with the JL and the JR diagnostic catheters being used in as much as 85% of the LCA angiograms and 75% of RCA angiograms, respectively.

The achievement of selective contrast injection into the coronary arteries, the most frequent arterial access approach (radial), and the most used catheter diameter (5 French) chosen by the performing interventional cardiologists did not vary significantly after TAVR. Nevertheless, both the dose area product and the volume of contrast used were remarkably higher in the pre-TAVR procedures, as a larger number of coronary angiogram views and either a ventriculography and/or an aortogram were more likely performed in such procedures. After TAVR, in only one case out of 28, no LCA injection was attempted while the same happened in 5 cases involving the RCA, due to known chronic occlusion of these vessels, resulting in a mean of 0.96 ± 0.58 diagnostic catheters used for RCA angiography after TAVR (Table 2).

### 3.2. Percutaneous Coronary Interventions

Post-TAVR PCIs were performed at a mean time of 603 ± 516 days.  Table 3 summarizes procedural indications, timing, and vessel/lesion characteristics. While pre-TAVR PCIs were performed mostly in stable circumstances, such as in asymptomatic/silent ischemia or stable angina patients, PCIs after TAVR were mainly performed in ACS, such as non-ST elevation myocardial infarction (NSTEMI) or unstable angina, a pattern similar to that observed for the CA analysis.

All 11 cases of pre-TAVR left-sided PCI were performed with a 3.0 or 3.5 extra backup guiding catheter (XB) although a single change of curve size was observed in two of these cases. Post-TAVR left-sided PCI also had a XB guiding catheter chosen for 7 of 9 cases. In three of these cases, a change of catheter curve from 3.5 to 3.0 was observed, and two of them ended up being managed with a JL 3.5 guiding catheter. Pre-TAVR RCA PCI was achieved in only 2 cases with either a JR or an AL guiding catheter. Following TAVR, 3 other RCA PCIs were performed, again, with the same type of catheters or a Barbeau guiding catheter. Lack of the need to change the initially chosen guiding catheter was observed in all post-TAVR RCA PCIs. Of note, 2 patients had both pre- and post-TAVR vein graft PCI in which either an AL, an AR, or an MP guiding catheter was used. Despite the changes observed in both catheter sizes and types, a significant increase in the total number of guides needed to perform PCI was not observed (pre-TAVR: 1.15 ± 0.38; post-TAVR: 1.54 ± 0.66; *p*=0.132).

Similar to the post-TAVR CA, no significant differences in achieving selective coronary injection or arterial access site preference (radial artery) were observed in post-TAVR PCI. Most guiding catheters had a 6F diameter before and after TAVR, with type B2 or C lesions present in the left coronary bed being the most common lesion type treated during the vast majority of PCI in both the pre- and post-TAVR periods. Other technical procedural aspects, namely, total procedural duration, fluoroscopy time, amount of contrast used and the dose area product also did not change after TAVR.

**Figure 3:**
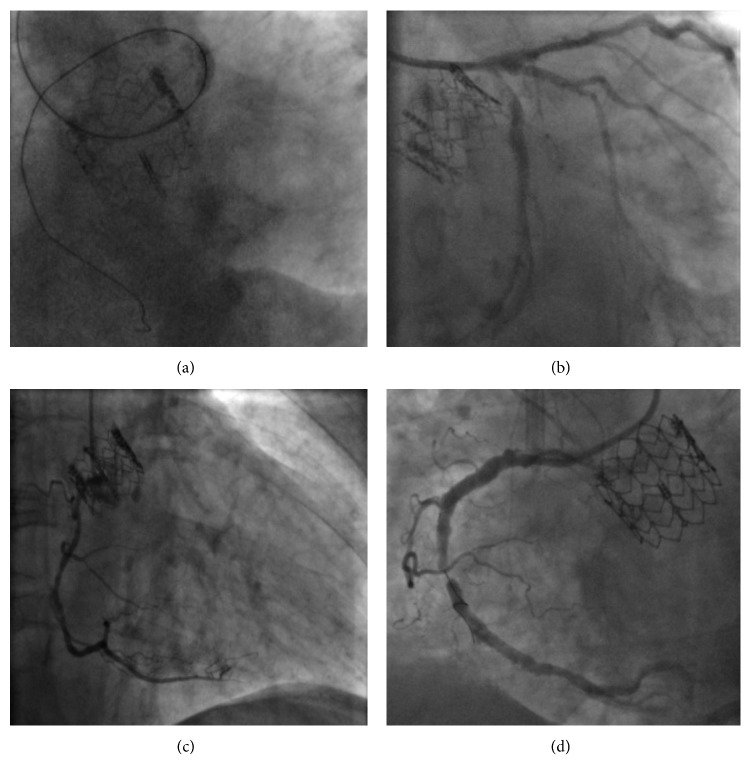
Post-TAVR PCI cases. (a) 5-French extra backup guiding catheter used during a PCI performed through the THV struts. (b) 6-French extra backup guiding catheter used during a PCI performed over the THV struts. (c) 5-French Judkins right guiding catheter used during a PCI performed through the THV struts. (d) 6-French Barbeau guiding catheter used during a PCI performed over the THV struts.

Although the type of stent, mainly drug-eluting stents, and number of lesions treated did not significantly differ between procedures, there was a significantly greater number of stents implanted pre-TAVR, which lead to a larger total stent length used in such procedures. Unlike what was observed during pre-TAVR PCI, no adjunctive instruments, such as the GuideLiner catheter (Vascular Solutions Inc., Minneapolis, MN, USA) or the rotational atherectomy system (Rotablator™, Boston Scientific, Marlborough, MA, USA), were used in the interventions after TAVR. Fluoroscopic/angiographic images of PCI before and after TAVR are shown in Figure 3.

## 4. Discussion

The present study demonstrates that CA and PCI performed following TAVR with a balloon-expandable Edwards valve did not exhibit significant changes in technical procedural aspects such as choice of arterial access site, catheter diameter, number of diagnostic or guiding catheters needed to perform the procedure, procedural duration, fluoroscopy time, contrast volume, and achievement of selective coronary injection compared with the respective pre-TAVR setting.

Aortic stenosis and coronary artery disease often coexist [1, 5–7], and updated international guidelines on coronary revascularization currently deem implementation of percutaneous coronary interventions on patients with symptomatic CAD prior to TAVR as appropriate [8, 9]. This practice has in fact long been adopted not only in our institution but also in most centers worldwide [7, 10–14]. The rationale for providing complete revascularization for impending TAVR candidates is based on the notion of lowering the risk from coronary ischemia that may transiently occur during the TAVR procedure *per se* (i.e., during rapid ventricular pacing and aortic balloon inflation) [7, 10, 12] along with improving symptoms and clinical outcomes post-TAVR.

However, a recent meta-analysis has suggested that revascularization pre-TAVR does not seem to award any clinical advantage and might actually be associated with an increased risk of major vascular complications and 30-day mortality [12]. More importantly, however, appears to be the acknowledgement that TAVR has been progressively implemented in lower surgical risk patients [15–21], who are frequently younger and might not display clinically relevant CAD yet, but who may require future CA or PCI [14]. Such observations have raised concerns regarding the feasibility and possible challenges encountered when performing post-TAVR CAs and PCIs and their relation to the different types of THVs.

It is estimated that 3 to 7% of TAVR recipients undergo CA or PCI at midterm follow-up post-TAVR [7, 10, 14, 22, 23]. Diverse indications for post-TAVR coronary procedures have been portrayed in literature and in a few cohorts, such as the one presented, acute coronary syndromes and development or progression of angina pectoris were the leading causes for such procedures [7, 10, 14, 24]. This observation is key for interpreting possible differences amongst coronary procedures performed before and after TAVR, as procedures performed in ACS patients aim to identify and treat culprit lesions only, while pre-TAVR procedures are frequently performed in stable patients potentially targeting a more complete revascularization. This may partially explain the higher number of stents and total stent length in the pre-TAVR PCI procedures. Interestingly, most PCI procedures after TAVR were secondary to an ACS, mainly non-ST elevation myocardial infarction.

Fundamental differences exist amongst the several types of commercially available THVs, and a detailed knowledge of such characteristics and the potential challenges they impose on future coronary interventions is paramount [7, 10, 14]. Valves with a stent frame extending beyond the coronary ostia, as the self-expanding CoreValve series (Medtronic, Minneapolis, Minnesota, USA), have their structure designed with a central concavity as well as open cells that permit selective coronary catheterization. Balloon-expandable valves, such as the SAPIEN valve series (Edwards Lifesciences, Irvine, California, USA), are most frequently positioned in a subcoronary position or just at the level of the coronary ostia, as was observed in our study, and as such are expected to have less of an impact on coronary catheterization [5, 7, 10, 23]. The aortoventricular angulation, obtained by measuring the angle between the horizontal plane and the aortic annulus [25], plays an important role in both the delivery of the THV and its relation to the coronary ostia, particularly in cases where a long THV is implanted [26]. Large angulations found in horizontal aortas might prevent the achievement of a perpendicular deployment of THVs with long stent frames, potentially leading to a more extensive interaction with the coronary ostia. The balloon-expandable valves used in our study, however, present a much shorter stent frame height (∼14 to 19 mm) and thus, are less likely to have a significant interaction with the coronary arteries due to the aortoventricular angulation. Moreover, as presented in Table 1, both the angiogram and PCI groups presented a mean aortoventricular angulation below the usual threshold determining a problematic THV positioning [25].

An increase in the number of diagnostic or guiding catheters, procedural duration, fluoroscopy time, or different choice of arterial access was not found during CA or PCI in our cohort of balloon-expandable valve recipients, as has been traditionally described in the context of self-expanding valve coronary procedures [7, 10, 14, 22]. Interestingly, the dose area product and contrast amount were actually smaller during post-TAVR CA performed in our cohort, contrary to what has been found in self-expanding valve cohorts [10, 14]. Probable reasons for such findings are that, as expected, ventriculography and/or aortogram were significantly less likely to be executed after TAVR and a lower number of angiographic views were performed after TAVR. Nonetheless, a trend of less-frequent achievement of coronary selective injections, similar to what is observed in some CoreValve series [7, 14], was present in this cohort.

Post-TAVR PCIs in patients with self-expanding valves have been primarily undertaken through the femoral access approach [10, 22]; still, the radial access approach to PCI has been the preferred route at our institution for over 2 decades. The present analysis demonstrates that such a predilection for the transradial access remained unchanged despite the presence of a prior balloon-expandable THV. Six-French systems remained the most commonly used guiding catheters diameters after TAVR [14, 22] although 5-French guiding catheters were numerically twice as frequent during post-TAVR PCI in the present study. Interestingly, the treated coronary lesions after TAVR were mainly situated within the left coronary bed both in our cohort and in previously reported studies [10, 14, 22, 23]. A significantly smaller number of stents, as well as total stent length during post-TAVR in our studied population, were observed. This observation might be explained by the commonly observed practice of performing PCI in all lesions deemed significant prior to the TAVR procedure [7, 10–14].

As previously mentioned, inherent THV characteristics must be acknowledged to better understand the possible challenges presented in post-TAVR coronary procedures. Yudi et al. [5] have recently proposed a useful algorithm for CA and PCI after TAVR. While JL and JR catheters are considered the primary choices for performing CA regardless of the THV type, particularities such as using the J-wire to enter the diamond in front of the coronary ostia and the possibility of using a JR4 to cannulate the LCA might be beneficial in CoreValve recipients. Failure to perform selective injections with these catheters should be followed by the use of guiding catheters, such as the XB/Voda Left/Femoral Left for SAPIEN recipients or the Ikari Right/Femoral Left/MP for CoreValve recipients, which allow for better support, particularly with the concomitant coronary wiring and the possibility to perform a balloon-assisted tracking or to use a guide extension catheter regardless of the THV type. No specific guiding catheter was assigned as the first choice for PCI in a balloon-expandable THV recipient; however, the XB guiding catheter may bend and should be maneuvered carefully. In CoreValve recipients, the JL for the LCA, or the JR for the RCA, is the preferred choice. Interestingly, the majority of the aforementioned suggestions are in accordance with our findings, in which the JL/JR catheters were most commonly used for post-TAVR CA and rare changes in guiding catheter choices were observed after the implantation of a balloon-expandable THV. The technical similarities observed in our procedures, performed prior to the publication of suggestions, might be due to the high level of expertise of the interventional cardiologists involved in our study, who have performed numerous CAs and PCIs over several years of practice, most with over a decade of experience in a high-volume institution.

Several limitations of the current analyses warrant consideration. Although instruments and equipment were equally available at the time of the compared procedures, as previously stated, there was a limited number of procedures available for comparison and they were not necessarily performed by the same interventional cardiologist; as such, despite having been performed by experienced interventional cardiologists from a single high-volume tertiary institution, one may argue that the level of expertise may vary amongst the performing physicians. Furthermore, the difference in the clinical scenarios in which the procedures were performed, namely, the greater prevalence of ACS on post-TAVR procedures, could have influenced some of the results since in such scenarios, there is less time for planning and choosing the most appropriate material to undertake both the CA and PCI. The intention to achieve a complete revascularization prior to TAVR is seldomly observed in ACS presented by TAVR recipients, where the objective is essentially to diagnose and treat the culprit lesion. Additionally, one must not disregard the retrospective nature of the present study and its inherent limitations.

## 5. Conclusion

In conclusion, following TAVR with a balloon-expandable Edwards-SAPIEN or SAPIEN XT valve, both CA and PCI are feasible and may be undertaken without substantial interaction with the implanted THV. 

## Figures and Tables

**Table 1:** Baseline clinical characteristics and TAVR procedural data from the coronary angiogram group and the PCI group.

	Angiogram group (*n* = 28)	PCI group (*n* = 13)
Clinical characteristics		
Age (years)	72.54 ± 9.08	70.31 ± 7.99
Male	14 (50.0)	9 (69.2)
Body mass index (kg/m^2^)	28.94 ± 5.96	28.63 ± 5.38
NYHA functional class III-IV	17 (60.7)	6 (46.2)
Diabetes	13 (46.4)	5 (38.5)
Hypertension	24 (85.7)	13 (100)
Coronary artery disease	23 (82.1)	13 (100)
Prior percutaneous coronary intervention	15 (53.6)	13 (100)
Prior coronary arterial bypass graft	14 (50.0)	9 (69.2)
History of atrial fibrillation	4 (14.3)	3 (23.1)
Peripheral vascular disease	9 (32.1)	7 (53.8)
Chronic obstructive pulmonary disease	7 (25.0)	5 (38.5)
Chronic kidney disease	15 (53.6)	10 (76.9)
eGFR (mL/min)	60.36 ± 24.63	58.47 ± 26.01
STS-PROM (%)	5.25 ± 2.71	5.89 ± 3.06
LogEuroSCORE (%)	17.22 ± 11.79	17.45 ± 12.72
Left coronary height (mm)	12.77 ± 3.27	12.32 ± 2.72
Right coronary height (mm)	13.23 ± 3.20	14.50 ± 4.59
Aortoventricular angulation (degrees)	42.40 ± 7.97	43.30 ± 7.50
Procedural characteristics		
Success	28 (100)	13 (100)
Approach		
Transfemoral	8 (28.6)	4 (30.8)
Nontransfemoral	20 (71.4)	9 (69.2)
Prothesis type		
Edwards-SAPIEN	12 (42.9)	6 (46.2)
SAPIEN XT	16 (57.1)	7 (53.8)
Prothesis size (mm)		
23	15 (53.6)	5 (38.5)
26	11 (39.3)	6 (46.2)
29	2 (7.1)	2 (15.4)
THV-coronary ostia position		
Supra-ostial	5 (17.8)	3 (23.0)
Ostial level	8 (28.6)	5 (38.5)
Infra-ostial	15 (53.6)	5 (38.5)

Variables are expressed as *n* (%) or mean ± SD. eGFR = estimated glomerular filtration rate; LogEuroSCORE = logistic EuroSCORE predicted risk of mortality; NYHA = New York Heart Association; STS-PROM = Society of Thoracic Surgeons Predicted Risk of Mortality; THV = transcatheter heart valve.

**Table 2:** Clinical and procedural characteristics for the coronary angiogram group (*n* = 28).

	Pre-TAVR	Post-TAVR	*p* value
Clinical characteristics			
Time from TAVR (days)	428.57 ± 1287.40	748.75 ± 686.62	—
Procedure indication			
Asymptomatic/silent ischemia	21 (75.0)	10 (35.7)	0.008
Stable angina	2 (7.1)	0 (0)	—
Unstable angina	3 (10.7)	11 (39.3)	0.021
Non-ST elevation myocardial infarction	2 (7.1)	5 (17.9)	0.257
ST elevation myocardial infarction	0 (0)	2 (7.1)	—
Procedural characteristics			
Access site			
Femoral	9 (32.1)	10 (35.7)	0.655
Radial	19 (67.9)	18 (64.3)	0.602
Catheter diameter (French)			
4	0 (0)	2 (7.1)	—
5	28 (100)	25 (89.3)	—
6	0 (0)	1 (3.6)	—
Number of diagnostic catheters used			
Left coronary artery	1.21 ± 0.69	1.04 ± 0.33	0.219
Right coronary artery	1.21 ± 0.79	0.96 ± 0.58	0.305
VG/LIMA	0.79 ± 0.96	0.82 ± 0.94	1.000
Number of guidings needed			
Left coronary artery	0.14 ± 0.36	0 ± 0	0.125
Right coronary artery	0 ± 0	0 ± 0	—
Selective injection achieved			
Left coronary artery	25 (92.6)	22 (81.5)	0.257
Right coronary artery	22 (95.7)	19 (82.6)	0.083
Number of angiographic views	12.27 ± 4.71	9.85 ± 3.03	0.004
Ventriculography ± aortogram performed	22 (78.6)	11 (39.3)	0.008
Procedural duration (min)	24.86 ± 10.85	23.43 ± 8.36	0.877
Fluoroscopy time (min)	7.59 ± 6.06	6.23 ± 4.20	0.269
Dose area product (cGy/cm^2^)	6295.88 ± 5125.99	4252.11 ± 2255.88	0.008
Contrast amount (mL)	125.32 ± 58.27	83.79 ± 40.77	0.003

Variables are expressed as *n* (%) or mean ± SD. LIMA = left internal mammary artery; VG = venous graft.

**Table 3:** Clinical, lesion, and procedural characteristics for the PCI group (*n* = 13).

	Pre-TAVR	Post-TAVR	*p* value
Clinical characteristics			
Time from TAVR (days)	37.31 ± 39.38	603.92 ± 516.58	—
Procedure indication			
Asymptomatic/silent ischemia	9 (69.2)	2 (15.4)	0.008
Stable angina	2 (15.4)	1 (7.7)	0.564
Unstable angina	2 (15.4)	4 (30.8)	0.414
Non-ST elevation myocardial infarction	0 (0)	6 (46.2)	—
ST elevation myocardial infarction	0 (0)	0 (0)	—
Procedural characteristic**s**			
Vessel treated			
Left main	6 (46.2)	2 (15.4)	0.046
Left anterior descending artery	5 (38.5)	4 (30.8)	0.564
Circumflex artery	7 (53.9)	4 (30.8)	0.083
Ramus intermedius	2 (15.4)	2 (15.4)	1
Right coronary artery	2 (15.4)	3 (23.1)	0.317
Venous graft	2 (15.4)	2 (15.4)	1.000
Lesion location			
Ostial	7 (53.9)	6 (46.2)	0.564
Bifurcation	7 (53.9)	4 (30.8)	0.180
Type B2/C lesion	12 (92.3)	11 (84.6)	0.564
Diameter stenosis (%)	83.17 ± 12.13	89.31 ± 11.88	0.267
TIMI flow before PCI			
0	1 (7.7)	1 (7.7)	1
1	0 (0)	1 (7.7)	—
2	1 (7.7)	2 (15.4)	0.564
3	11 (84.6)	9 (69.2)	0.414
TIMI flow after PCI			
0	0 (0)	0 (0)	—
1	0 (0)	1 (7.7)	—
2	0 (0)	0 (0)	—
3	13 (100)	12 (92.3)	—
Access site			
Femoral	3 (23.1)	4 (30.8)	0.655
Radial	10 (76.9)	8 (61.5)	0.414
Both	0 (0)	1 (7.7)	—
Catheter diameter (French)			
5	3 (23.1)	6 (46.2)	0.257
6	8 (61.5)	7 (53.89)	0.655
7	2 (15.4)	0 (0)	—
Number of guidings needed	1.15 ± 0.38	1.54 ± 0.66	0.132
Selective injection achieved	12 (92.3)	13 (100)	—
Number of lesions treated	1.85 ± 0.90	1.31 ± 0.48	0.180
Number of stents implanted	2.31 ± 1.55	1.31 ± 0.75	0.037
Total stent length (mm)	57.23 ± 46.65	25.69 ± 18.06	0.018
Maximum stent diameter (mm)	3.15 ± 0.47	3.13 ± 1.07	0.680
Type of stent used			
Bare-metal stent	3 (23.1)	1 (7.7)	0.317
Drug-eluting stent	10 (76.9)	11 (84.6)	0.655
Balloon predilation	13 (100)	11 (84.6)	—
Balloon postdilation	11 (84.6)	9 (69.2)	0.414
GuideLiner used	1 (7.7)	0 (0)	—
Rotational atherectomy performed	4 (30.8)	0 (0)	—
Aspiration thrombectomy	0 (0)	1 (7.7)	—
Number of angiographic views	16.46 ± 7.97	15.07 ± 6.00	0.667
Fluoroscopy time (min)	20.99 ± 20.92	20.69 ± 11.94	0.677
Procedural duration (min)	63.15 ± 37.41	65.23 ± 26.38	0.672
Dose area product (cGy/cm^2^)	13166 ± 8056	8098 ± 5579	0.131
Contrast amount (mL)	170 ± 86.98	148.92 ± 58.85	0.748
Procedural success	13 (100)	12 (92.3)	—

Variables are expressed as *n* (%) or mean ± SD. PCI = percutaneous coronary intervention.

## Data Availability

The data used to support the findings of this study are available from the corresponding author upon request.
